# Assessment and mitigation of geometric distortions in Cartesian MR images at 15.2T for preclinical radiation research

**DOI:** 10.1002/mp.17963

**Published:** 2025-07-14

**Authors:** Silvia Stocchiero, Isselmou Abdarahmane, Esaú Poblador Rodríguez, Vanessa Fröhlich, Markus Zeilinger, Dietmar Georg

**Affiliations:** ^1^ Competence Center for Preclinical Imaging and Biomedical Engineering, Faculty of Health University of Applied Sciences Wiener Neustadt Austria; ^2^ Department of Radiation Oncology Medical University of Vienna Vienna Austria; ^3^ MedAustron Ion Therapy Center Wiener Neustadt Austria

**Keywords:** geometric distortion, small‐animal radiotherapy, ultra‐high field MRI

## Abstract

**Background:**

Ultra‐high field (UHF) magnetic resonance (MR) systems are advancing in preclinical imaging offering the potential to enhance radiation research. However, system‐dependent factors, such as magnetic field inhomogeneities ($\Delta B_{0}$) and gradient non‐linearity (GNL), induce geometric distortions compromising the sub‐millimeter accuracy required for radiation research.

**Purpose:**

This study tackles system‐dependent distortions in 15.2T MR images by prospective shimming strategies optimization and comparing two imaging methods for voxel displacement correction. The methods were evaluated on a 3D‐printed grid phantom and validated on in vivo mouse brain MR images. Additionally, a phantom‐based displacement map was tested for GNL correction in mouse brain images.

**Methods:**

Phantom MR and CT images were acquired with 200$\mu m^{3}$ resolution. In vivo mouse brain MR and CT images had 140$\mu m^{3}$ and 200$\mu m^{3}$ resolutions, respectively. Three shimming strategies were established to assess $\Delta B_{0}$ displacements ($\Delta r_{B_{0}}$) in phantom MR images. $\Delta r_{B_{0}}$ was calculated using the acquired static field maps in three volumes of interest (VOIs) via Python script. A one‐step distortion correction (1SDC) method, which simultaneously corrects $\Delta B_{0}$ and GNL distortions via non‐rigid registration with CT, and a two‐step distortion correction (2SDC) method, which corrects separately in two consecutive steps $\Delta B_{0}$ and GNL displacements, were assessed on phantom and in vivo images. For in vivo 2SDC validation, a phantom displacement map generated by MR to CT non‐rigid registration was applied to correct GNL on the mouse brain. Total displacements ($\Delta r_{tot}$) were quantified in phantom VOIs and the in vivo skull region by measuring landmarks' positions.

**Results:**

The $\Delta r_{B_{0}}$ in the phantom increased with distance from the VOI center and magnet isocenter. Shimming scenario‐2 showed the lowest maximum displacement (0.26 mm) for the largest VOI but required a longer acquisition time. Distortion correction methods were necessary for large VOIs (13–25 mm, along the *z*‐axis) in the phantom where $\Delta r_{tot}$ >0.2 mm. The 2SDC method outperformed 1SDC by achieving a ≤0.2 mm accuracy in 100%, 92.1%, and 59.3% of the landmarks from the smallest to the largest VOI. Phantom dice scores confirmed the improvement in geometric precision after each correction step. In vivo results showed that 1SDC correction overcorrected MR images, increasing voxel displacements. The 2SDC exceeded the 1SDC, reducing $\Delta r_{tot}$ by 85%, in accordance with the dice score analysis (0.97 2SDC vs. 0.84 1SDC).

**Conclusions:**

At 15.2T, in vivo MR images of even small regions (e.g., mouse brain) require geometric distortion correction for radiation research. The 2SDC method outperformed the 1SDC, emphasizing the need for separate $\Delta B_{0}$ and GNL corrections. Moreover, a phantom‐based displacement map shows promise for in vivo GNL correction.

## 1 INTRODUCTION

Magnetic resonance imaging (MRI) is a powerful and versatile technique with superior soft tissue contrast for non‐invasive tissue characterization compared to commonly used computed tomography (CT), yielding valuable complementary information for assessing tumor aggressiveness and radiation effects in vivo.^1^, ^2^, ^3^ Especially in the context of radiation research with animal models, MRI offers unique opportunities for longitudinal studies. Specifically, in ion beam therapy (IBT), further investigations are essential to elucidate tissue response as a function of dosimetric parameters, such as absorbed dose and linear energy transfer (LET). Small animal models and a preclinical image‐guided irradiation workflow with MR‐based response assessment could provide new insights in this respect.

Ultra‐high field (UHF) MR systems, operating at field strengths beyond 9.4T and up to 18T, are rapidly advancing in preclinical research. Their higher sensitivity and spatial resolution yield the potential to enhance radiation research.^4^, ^5^, ^6^, ^7^ Preclinical MR scanners are designed with narrower bores, shorter coils, and higher gradient strengths than in clinical systems. This comes with technical challenges and drawbacks related to each hardware component, inhomogeneities of the static ($B_{0}$) and transmit ($B_{1}$) magnetic fields, as well as gradient non‐linearity (GNL).

It is widely recognized that MR acquisitions suffer from geometric image distortion, which becomes even more pronounced when utilizing UHF systems.^7^, ^8^, ^9^, ^10^ In the frame of radiotherapy, distortions pose a challenge when aiming to correlate dosimetric parameters with morphological observations or imaging biomarkers based on MR images. Therefore, the evaluation and understanding of spatial distortion allow for its correction in MR images, enabling their use in treatment planning for organ contouring and tissue characterization, which is essential to improve preclinical radiation research.^11^, ^12^ MRI geometric distortions can be generally categorized as system‐dependent, caused by device inaccuracies, or patient‐dependent, resulting from the subject's magnetic features.^7^, ^8^, ^9^

Chemical shift and magnetic susceptibility are the main contributing effects to patient‐dependent distortions. The latter arises from differences in the magnetic properties of tissues, leading to local variations in the magnetic field, while chemical shift results from variations in magnetic shielding across chemical environments.^7^, ^8^, ^13^ Magnetic field inhomogeneities ($\Delta B_{0}$) and GNL represent the primary system‐dependent sources of geometric errors in MR images and are also the most dominant ones.^7^, ^8^ Both system‐dependent distortions become accentuated with increasing distance from the scanner isocenter (i.e., large field of views ‐ FoV). $\Delta B_{0}$ effects are amplified by both high field strengths and short bores, while GNL are accentuated particularly in fast gradient sets using short coils.^7^, ^13^, ^14^ The distortion measured in standard Cartesian MR images results in a displacement of the voxel from its initial position. GNL induces in these images voxel displacement in all three spatial directions (x, y, and z), and its distortions are sequence‐independent, remaining constant across scans and subjects.^8^, ^9^ Conversely, the $\Delta B_{0}$ cause sequence‐dependent distortions influencing the frequency encoding direction, also leading to signal loss.^7^, ^8^, ^9^ Distortions in target proximity spread directly into uncertainties for voxel‐based response assessment or even when aiming to correlate dosimetric information with radiologic findings in small structures. This indicates how essential and challenging the geometric distortion correction is for radiotherapy applications, especially when utilizing UHF scanners.

The present study introduces in‐house developed quality assurance (QA) methods employing a 3D‐printed grid phantom to evaluate and correct system‐dependent geometric distortions in Cartesian MR images at 15.2T. The first aim of this investigation was to assess and mitigate $\Delta B_{0}$ distortions depending on the active shimming strategy for different volumes of interest (VOI) sizes in the grid phantom. Active shimming is a commonly used procedure to adjust the magnetic field homogeneity in MRI, typically performed automatically with first‐order global shimming to correct linear variations. Higher‐order (2nd, 3rd) shimming is often applied locally and addresses quadratic and cubic variations to mitigate significant $\Delta B_{0}$ distortions. This analysis evaluates the 15.2T scanner's shimming performance limits, using the grid phantom as a benchmark by identifying the maximum volume requiring $\Delta B_{0}$ correction.

As a second objective, total geometric distortions characterized mainly by $\Delta B_{0}$ and GNL were quantified and corrected using two distinct in‐house developed imaging methods on the grid phantom. Geometric distortion correction methods mostly use polynomial‐based global transformations (e.g., non‐rigid registrations) applied to MR images, using non‐distorted CT as the reference.^4^, ^8^ In clinical applications, specific GNL correction can be achieved using the spherical harmonic expansion method, with vendors providing the necessary coefficients to calculate the GNL displacements.^17^ However, preclinical scanners often lack the software tools to provide these coefficients, requiring users to develop complex correction algorithms themselves. This aspect is further complicated by hardware limitations, lack of standardization, and additional costs for tool development. In standard QA, distortions can be measured before and after correction using grid phantoms.^4^, ^7^, ^8^, ^9^, ^10^, ^13^, ^14^, ^15^, ^16^, ^18^ The grid intersection points serve as landmarks, allowing their 3D positions to be compared between MR and CT. In this study, first, a one‐step distortion correction (1SDC) method, employing non‐rigid registration with CT images, was used to simultaneously address both $\Delta B_{0}$ and GNL distortions. Second, a two‐step distortion correction (2SDC) approach was implemented, first rectifying voxel displacements caused by $\Delta B_{0}$ followed by non‐rigid registration to correct GNL distortions. A phantom displacement map was generated after non‐rigid registration.

The final aim involved the validation of the two proposed imaging methods on mouse brain images. Brain MR images pose distortion correction challenges in the areas close to air cavities, with strong soft tissue‐air boundaries. In the in vivo 2SDC, the usage of a phantom displacement map was evaluated to correct GNL distortion. Assuming that GNL is sequence‐independent, related voxel displacements in the in vivo MR image could be corrected directly by applying shift values in each space direction driven by the phantom displacement map.

## 2 SYSTEM‐DEPENDENT DISTORTIONS

In standard anatomical 3D MR imaging based on spin‐echo (SE) or gradient‐echo (GRE) acquisitions, commonly referred to as Cartesian sequences, the spatial position v(x,y,z) of a given voxel is shifted in position $v^{\prime }(x^{\prime },y^{\prime },z^{\prime }$) due to an in‐plane displacement $\Delta r_{tot}$. This displacement comprises two primary system‐dependent contributions: $\Delta B_{0}$ distortions ($\Delta r_{B_{0}}$) and gradient non‐linearity ($\Delta r_{GNL}$), as described in Equation (1) below:^7^, ^8^, ^9^

(1) $$v^{\prime }=v+\Delta r_{tot}=v+\Delta r_{B_{0}}+\Delta r_{GNL}$$

According to the literature, in Cartesian acquisitions, as considered in this study, $\Delta B_{0}$ distortions are sequence‐dependent and exclusively impact the frequency encoding direction, with no effect on the phase encoding or slice selection directions. In contrast, GNL induces voxel displacements in all three spatial directions (x,y,z) and is sequence‐independent.^7^, ^8^, ^9^ Consequently, every image acquired on a given scanner is subject to the same GNL distortion, stemming from the inherent non‐linearity of the gradient coils in the x, y, and z directions. In Equation (1), eddy‐current‐related effects were not considered, as they are negligible for Cartesian MR sequences. For a Cartesian 3D MR image acquired with the frequency encoding direction along the z‐axis, while the phase encoding and slice selection directions are set along x and y, respectively, the combined $\Delta B_{0}$ and GNL‐related distortion contributions can be expressed as follows by reformulating Equation (1):

(2) $$\begin{array}{cc} x^{\prime } & =x+\frac{\Delta B_{G_{x}}\left(x,y,z\right)}{G_{x}}\\ y^{\prime } & =y+\frac{\Delta B_{G_{y}}\left(x,y,z\right)}{G_{y}}\\ z^{\prime } & =z+\frac{\Delta B_{0}\left(x,y,z\right)}{G_{z}}+\frac{\Delta B_{G_{z}}\left(x,y,z\right)}{G_{z}} \end{array}$$

where ($x^{\prime },y^{\prime },z^{\prime }$) are the coordinates of the voxel in the position affected by distortions, (x,y,z) correspond to the non‐shifted positions, $\Delta B_{G_{x}}$, $\Delta B_{G_{y}}$, $\Delta B_{G_{z}}$ are displacements due to the GNL in each direction, $G_{x},G_{y},G_{z}$ are the gradient strengths, and $\Delta B_{0}$ represents the displacement due to the static field inhomogeneity.^7^, ^8^, ^9^, ^15^, ^19^

## 3 MATERIALS AND METHODS

### 3.1 3D grid phantom and holder design

**FIGURE 1:**
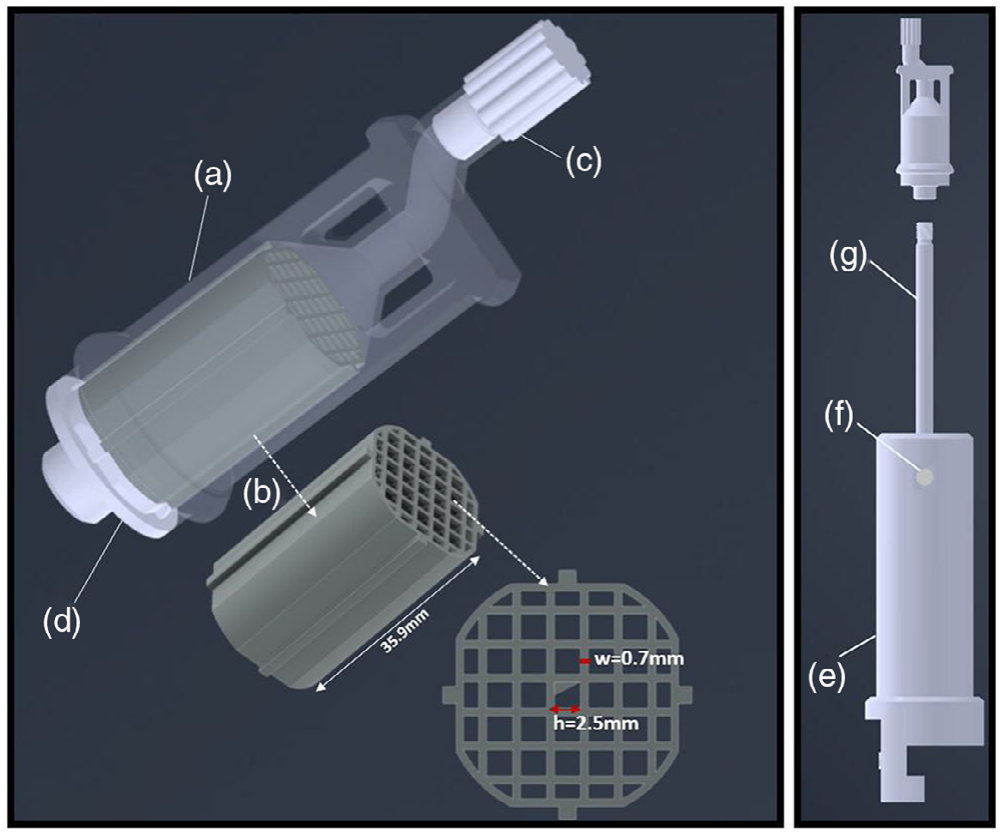
CAD file representations of the 3D‐printed phantom components (left) and phantom holder (right): (a) external structure, (b) cubic grid structure, (c) top cap, (d) bottom cap, (e) holder body, (f) screw, and (g) threaded rod. CAD, computer‐aided designs.

A customized grid phantom was designed and in‐house manufactured, using the prototype developed by O'Challaghan et al. (tailored for a 9.4T/31 cm bore‐diameter Agilent scanner) as a reference.^4^ SOLIDWORKS software (Dassault Systèmes, France) was used to create computer‐aided designs (CAD) for the phantom and the holder. The phantom was 3D printed in plastic powder polyamide‐12 (PA 2200 Material Data Sheet), a nylon material with magnetic susceptibility similar to water optimal for MRI studies, using an EOS P396 laser printer system (EOS, Germany). Figure 1 shows the four phantom components and the holder. The dimensions of the external structure (a) are 28×28×78 $mm^{3}$ and the ones of the detachable inner cubic grid (b) are 22×22×35.6 $mm^{3}$ with a cube wall thickness of 0.7 mm (w) and gap (h) distances of 2.5 mm. The central region of the grid features an asymmetric triangle strategically integrated to aid image alignment. Two distinct caps (c,d) were fabricated to seal the phantom edges. The lower edge cap incorporated a screw mechanism, facilitating attachment to the holder and reproducible phantom positioning into the coil. The phantom holder was manufactured in white POM‐C using conventional machining. It consists of a rigid cylinder (e) of size 5.5×5.5×21.8$\;cm^{3}$ in which a rod (g) with a threaded top (70 mm diameter) can be inserted. First, the rod was machined on a lathe, its length can be adjusted with a screw (f). The borings and the fixation were performed with a milling machine.

### 3.2 Imaging

**FIGURE 2:**
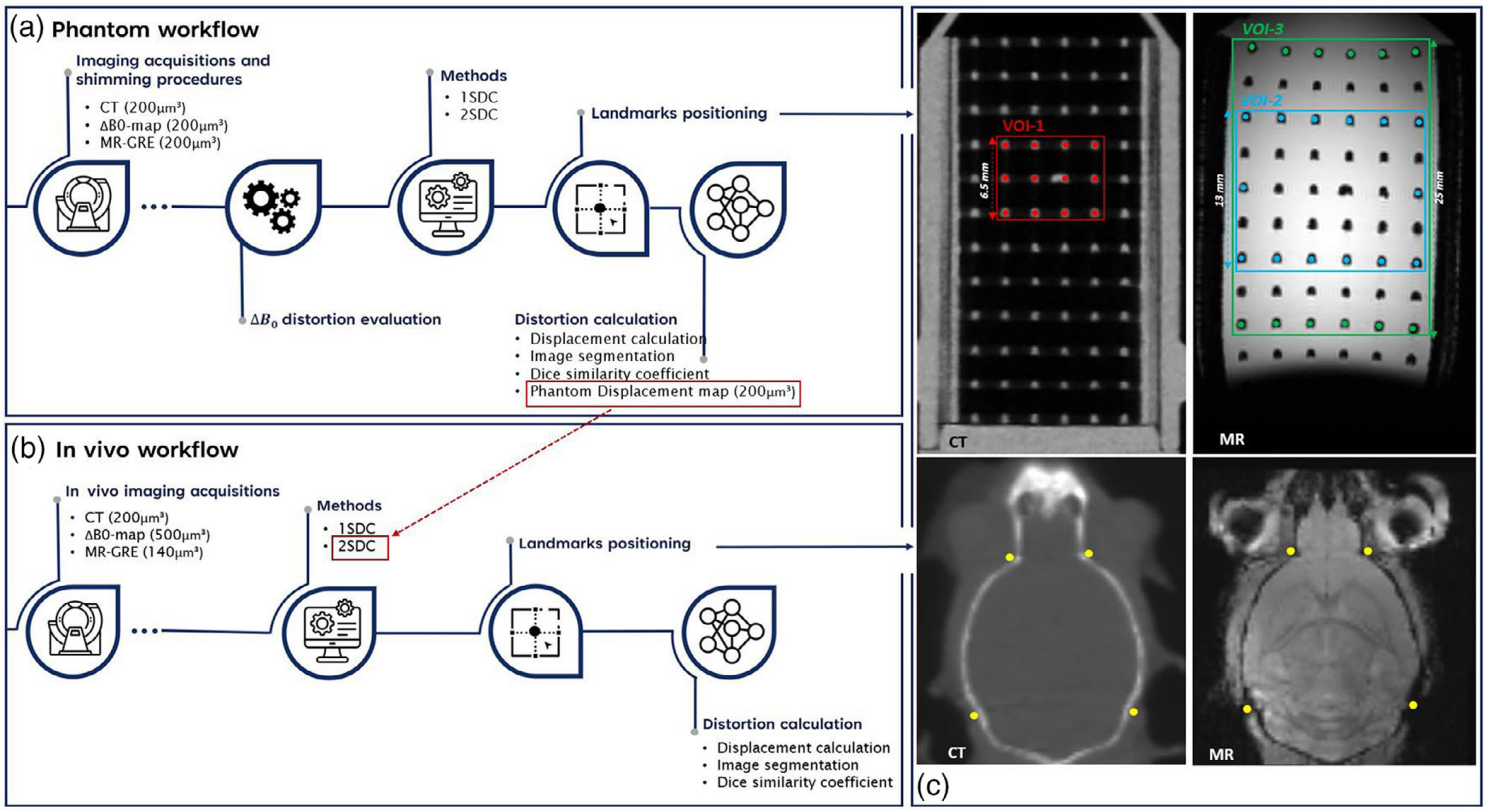
Flowchart of the phantom (a) and in vivo (b) workflows. (c) CT and MR images of the grid phantom (top) and mouse brain (bottom) with position landmarks for the different VOIs and around the skull. Central coronal slices of CT and MR images from left to right. VOIs, volumes of interest.

Figure 2a,b illustrate the flowchart of the proposed phantom and in vivo workflows, from image acquisition and shimming applications to the post‐processing steps applied for distortion evaluation.

#### 3.2.1 Phantom acquisitions

CT acquisitions were performed with a spiral cone beam CT (X‐CUBE; Molecubes, Belgium) for which the grid phantom was kept empty (filled with air) to ensure the phantom material visibility in the image. A FoV of 70×70×54.6 $mm^{3}$ was selected for covering the grid region, with 200 $\mu m^{3}$ resolution. The CT images and the grid positions were considered as ground truth for assessing MR distortions. MR images were acquired with a 15.2 T/11 cm bore‐diameter Biospec MRI scanner (Bruker BioSpin, Germany) equipped with a gradient system characterized by a maximum gradient strength of 1000 mT/m, slew rate of 10000 T/m/s, minimum rise time of 300 μs and 60 mm internal diameter, coupled with a quadrature RF volume coil with 3.5 cm inner diameter. Before MR imaging, the phantom was filled with an in‐house prepared copper(II) sulphate solution (1 mg/mL) to enhance the visibility of the gaps between the grids. The following coronal 3D acquisitions were utilized for the purpose of this study: static field ($\Delta B_{0}$) maps based on double‐echo GRE sequence (TR/TE = 11.2/2.32 ms; FA = $10^{\circ }$; NA = 2) and a GRE sequence (TR/TE = 17/4 ms; FA = 10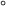; NA = 2) with a FoV of 30×30×42 $mm^{3}$ and 200 $\mu m^{3}$ resolution. For each MR scan, the z longitudinal orientation was set as frequency encoding direction (parallel to the bore of the magnet) using a readout gradient strength ($G_{z}$) of 56.1 mT/m and a receiving bandwidth (BW) of 100 kHz. The $\Delta B_{0}$ map acquisition time was 16.5 min, for GRE 14.2 min. Figure 2 shows the resulting CT and MR images of the grid phantom.

#### 3.2.2 In vivo acquisitions

Female BALB/cJRj mice underwent CT and MR acquisitions (Ethics approval from the Federal Ministry of Education, Science and Research of Austria: 2023‐0.556.474). Mice were anaesthetized with 1%–3% isoflurane and physiological parameters were monitored during imaging acquisitions. CT images of the animals were acquired with 200 $\mu m^{3}$ resolution. The MR setup was equipped with a surface mouse head coil with a 23 mm inner diameter. A $\Delta B_{0}$ map (FoV = 32×32×32 ${\mathrm{mm}}^{3}$; TR/TE = 10/1.54 ms; FA = 10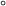; NA = 2) with 500$\mu m^{3}$ resolution and a coronal T1‐weighted GRE (FLASH) 3D sequence (FoV = 18×18×15.7 ${\mathrm{mm}}^{3}$; TR/TE = 90/3.5 ms; FA = 17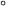; NA = 1) with 140 $\mu m^{3}$ resolution covering the mouse head, were selected in z frequency encoding direction.

Saturation bands were applied to reduce artifacts and inhomogeneities due to air‐soft tissue differences in the ear canals. All in vivo MR images, including the $\Delta B_{0}$ map, were optimized in the skull area by implementing additional static field map‐based shimming (up to the 3rd‐order) to improve homogeneity. In this study, CT and MR images from only one mouse were used (Figure 2c).

### 3.3 Phantom‐based $\Delta B_{0}$ distortion quantification and shimming optimization

The following three shimming strategies were established as part of scanner‐specific shimming commissioning to quantify and optimize $\Delta B_{0}$ distortions utilizing the grid phantom:

***Shimming scenario‐1*** (**Shim‐1**): linear 1st‐order shimming with initial shimming values set to zero.

***Shimming scenario‐2*** (**Shim‐2**): scenario‐1 with additional automatic estimation of all shimming values up to the 3rd‐order based on pre‐scanned $\Delta B_{0}$ maps generated in a pre‐scan (*MapShim —ParaVision7.0*).^19^

***Shimming scenario‐3*** (**Shim‐3**): scenario‐1 with pre‐defined optimized initial shimming values including all 2nd‐order shimming coefficients and the $z_{3}$ coefficient.

The MR protocol—including static field maps ($\Delta B_{0}$) and GRE sequences—was performed three times for each shimming scenario without repositioning the phantom. A comparison of the $\Delta B_{0}$ maps acquired after the application of each shimming scenario is reported in Appendix (A). $\Delta B_{0}$ maps account for system‐dependent $\Delta B_{0}$ distortions and patient‐induced susceptibility effects, which influence the frequency encoding direction. However, with the phantom's homogeneous composition (Section 3.1), susceptibility effects can be disregarded in this context.^7^, ^19^ The displacement ($\Delta r_{B_{0}}$), arising from $\Delta B_{0}$ along the z readout encoding direction, was computed at a voxel‐level for the different shimming scenarios, according to Equation (3) as described in Section 2:^8^, ^9^, ^15^, ^19^

(3) $$\Delta r_{B_{0}}=\frac{\Delta B_{0}\left(x,y,z\right)}{G_{z}}$$

where $\Delta B_{0}\left(x,y,z\right)$ corresponds to the static field inhomogeneity present in each voxel of the $\Delta B_{0}$ map and $G_{z}$ is the gradient strength along z as the frequency encoding direction.^8^, ^9^, ^15^, ^19^ The voxel displacements were calculated via a custom Python script and repeated for each acquired static field map—for statistical robustness of the results—and each shimming scenario, thus enabling a comparison of shimming strategies. Three distinct volumes of interest (VOI) were defined within the phantom's grid and centered around the same origin with the following sizes, using the same script: 10×10×6.5$\;mm^{3}(VOI_{1}$), 16×16×13$\;mm^{3}(VOI_{2}$), and 16×16×25$\;mm^{3}(VOI_{3}$) (Figure 2b). These VOI dimensions were selected to evaluate $\Delta r_{B_{0}}$ across small, medium, and large FoVs by increasing distance from the VOIs' and the magnet's center.^7^, ^8^, ^9^ To mitigate strong signal loss and asymmetry along the z‐direction, the VOI centers were automatically positioned 2 mm higher than the magnet's isocenter by a script's command, thereby avoiding pronounced inhomogeneities. In our evaluation, only the additional voxels introduced by expanding VOI sizes were considered in the $\Delta r_{B_{0}}$ calculation. Therefore, after selecting and positioning the VOIs, the script excluded from $VOI_{2}$ the voxels included in $VOI_{1}$, as well as those in $VOI_{1}$ and $VOI_{2}$ from $VOI_{3}$. This methodology resulted in the following VOIs: VOI1, $VOI2_{-VOI1}$ and $VOI3_{-VOI2}$. Consequently, the distortions in peripheral regions farther away from the VOIs' center along the *z*‐axis were evaluated, where the largest displacements are expected based on existing literature.^4^, ^7^, ^8^, ^9^

### 3.4 Distortion correction methods

Two in‐house developed post‐processing imaging pipelines were set up and compared to reduce geometric distortions to the greatest extent feasible, utilizing commonly available software tools. Both pipelines were tested on the grid phantom to assess their efficiency in correcting geometric distortions at 15.2T under controlled conditions. In the next step, the pipelines were validated on in vivo mouse brain MR images, representing a more complex and realistic preclinical research scenario.

#### 3.4.1 Phantom

***1SDC***: This method assesses the total system‐dependent distortion ($\Delta r_{tot}$), arising from both GNL and $\Delta B_{0}$, and corrects both distortion types simultaneously using non‐rigid registration with the CT image. This approach represents a rapid and widely used distortion correction procedure in clinical MR‐QA for radiotherapy applications and was, therefore, selected for this study as the basis for a 15.2T distortion correction methodology.^4^, ^14^, ^15^ Non‐rigid registration transformation locally deforms the MR image to align with the reference CT, adjusting voxel positions ($v^{\prime }$) closer to their original locations (v) (Equation 1). However, it does not independently correct each system‐induced source of distortion, not distinguishing between those and true anatomical features, but rather compensating for their effects. Therefore, $\Delta r_{B_{0}}$ and $\Delta r_{GNL}$ displacements are not individually tackled. Depending on the non‐rigid registration algorithm's accuracy and due to the high degrees of freedom, this method may introduce overcorrection and misalignment, particularly in challenging images presenting artifacts or anatomical areas with pronounced tissue‐air interfaces. In this pipeline, the CT volume undergoes cropping using the ModelCropper plug‐in of 3D‐Slicer,^20^ to align with the dimensions of the MR volume. Subsequently, DICOM files are converted into the NIfTI format. *NiftyReg* software was employed for CT‐MR image registration.^21^, ^22^

For phantom images, first, the *reg_aladin* algorithm from *NiftyReg* was applied for rigid image registration of the MR images (*moving‐image*) to CT (*fixed‐image*), which aligns the two volumes by establishing spatial correspondences via block matching and leveraging normalized cross‐correlation to assess multimodal similarity. It is designed to reduce directional bias effectively and optimize transformations within the native image spaces, ensuring accurate alignment in the absence of landmarks.

Non‐rigid registration to CT images was executed on the rigid‐registered MR images (*moving*) applying the *reg_f3d* algorithm in *NiftyReg* to CT image (*fixed*), employing cubic B‐splines for deformation. The reg_f3d algorithm used a grid of control points for local adjustments, optimizing the alignment through normalized mutual information and bending energy within a conjugate gradient framework. The weight of the bending energy was set to 0.01. The grid spacing for the splines was selected as 10,10,10. A maximum number of 1000 iterations for the objective function were set.

***2SDC***: This approach was designed as an alternative to the 1SDC to fulfil the geometric accuracy requirements for radiotherapy purposes. The evaluation and correction of $\Delta B_{0}$ and GNL were performed separately in two consecutive steps. This sequential approach, in which a single displacement direction is independently corrected for $\Delta B_{0}$ as a first step, can improve the initial position of each voxel before the following correction step based on non‐rigid registration. From the acquired static field map of the phantom, a $\Delta B_{0}$‐spatial map was generated via a respective Python script containing the $\Delta r_{B_{0}}$ voxel displacements in millimeters, calculated using formula 3 (Section 3.3). The $\Delta B_{0}$‐spatial map was then applied to the original MR phantom image to correct for $\Delta B_{0}$ distortions by shifting voxel positions along the z readout encoding direction according to the $\Delta r_{B_{0}}$ displacements indicated in the $\Delta B_{0}$‐spatial map. Consequently, voxels were repositioned to compensate for $\Delta r_{B_{0}}$ displacements using the same script, resulting in a $\Delta B_{0}$‐corrected MR image, while the $\Delta r_{GNL}$ component still required correction as described in Section 2. Next, a rigid registration of the $\Delta B_{0}$‐corrected MR image (*moving*) to the CT image (*fixed*) was performed. The residual distortions, which are assumed to be primarily from GNL (Equation 1), were corrected via non‐rigid registration with the CT image (*fixed*) by generating a final $\Delta B_{0}$+Non‐rigid corrected MR image. A phantom displacement map with 200 $\mu m^{3}$ resolution, which contained $\Delta r_{GNL}$, was generated after non‐rigid registration. The same algorithms and parameters for rigid and non‐rigid registrations used in the 1SDC pipeline were also applied in this approach. Despite the potential advantages of the 2SDC method, this implies multiple steps, longer implementation time compared to the 1SDC, as well as the acquisition of a static field map for each MR image that has to be corrected.

#### 3.4.2 In vivo

***1SDC***: The same procedures and algorithms were used to evaluate and mitigate distortions in the in vivo MR images. The in vivo MR image was first downscaled to the in vivo CT resolution (200 $\mu m^{3}$) before rigid and non‐rigid registrations.

***2SDC**:* the 2SDC phantom procedure was repeated using CT as the *fixed* image and MR as the *moving* one to set the phantom displacement field map on the MR image reference system. The phantom displacement field was then upsampled from 200 $\mu m^{3}$ to the in vivo MR resolution (140$\;\mu m^{3}$). In parallel, the 2SDC method was performed on the in vivo images. First, the mouse brain $\Delta B_{0}$ map was upsampled from 500$\;\mu m^{3}$ to the in vivo MR image resolution and used to generate a mouse brain $\Delta B_{0}$‐spatial map using an in‐house developed Python script. A mouse brain MR $\Delta B_{0}$‐corrected image was obtained by applying the mouse brain $\Delta B_{0}$‐spatial map on the original MR volume via the same script. Then, the phantom displacement map (Section 3.4.1) containing the $\Delta r_{GNL}$ was applied on the mouse brain MR $\Delta B_{0}$‐corrected one via a second custom Python script to correct specifically for GNL and validate the non‐subject dependency of this distortion source (Figure 2b). In this case, the voxel positions in the in vivo MR image, which were already corrected by the $\Delta B_{0}$ distortions, are further shifted in each spatial direction (x,y,z) according to the $\Delta r_{GNL}$ displacements indicated in the phantom displacement map by correcting the image also for the GNL component in a direct way. This is possible by assuming that the GNL distortion is sequence‐independent, contrary to $\Delta B_{0}$ distortions, as already described in Section 2. Both the in vivo MR $\Delta B_{0}$‐corrected and the final $\Delta B_{0}$+GNL corrected images were downsampled to the CT image resolution (200$\;\mu m^{3}$) and rigidly registered to this latter, used as the reference (*fixed*) image for $\Delta r_{tot}$ evaluation. The two in‐house developed Python scripts, mentioned in this current section for $\Delta B_{0}$ and GNL distortion corrections, used to implement the in vivo 2SDC pipeline are publicly available online https://github.com/isselmou92/DistortionCorrection.git.

### 3.5 Quantification of total geometric distortions

#### 3.5.1 Phantom

The total system‐dependent distortion ($\Delta r_{tot}$), arising from both GNL and $\Delta B_{0}$, was assessed and verified by landmarks manually positioned by three different observers on both the CT and MR volumes. The $\Delta r_{tot}$ displacements were quantified in millimeters before and after the corrections of the 1SDC and 2SDC pipelines, to assess the residual distortions that could not be corrected. The landmarks were defined using 3D‐Slicer within three coronal and sagittal slices covering the three different VOIs with same size and position of those used for the evaluation of geometrical distortions from $\Delta B_{0}$ (Section 3.3). The landmarks were placed at the intersections of the phantom lattice. For VOI2 and VOI3, they were located only at the edges of the volume to assess displacement with increasing distance from the VOIs' center (Figure 2c), therefore resulting in $VOI2_{-VOI1}$ and $VOI3_{-VOI2}$ as described in Section 3.3. Each observer placed 36 landmarks for VOI1 and $VOI3_{-VOI2}$, and 42 for $VOI2_{-VOI1}$. Areas affected by signal loss due to transverse relaxivity‐$R_{2}$ and signal loss in GRE images were excluded from the VOIs contouring. The final set of landmarks was then exported from 3D‐Slicer to an in‐house developed Python script to calculate Euclidean distances as well as directional displacements in x,y, and z axes between corresponding CT and MR based landmarks. Phantom automatic segmentation of the CT and co‐registered MR volumes was performed in 3D‐Slicer for the whole region of VOI3, employing Otsu's automatic thresholding method.^23^ Threshold values were set at ‐835.04, ‐401.83 for CT images and 0.03, 0.59 for MR images. Image processing of the phantom segmented 3D label map volumes of the different VOIs from the grid phantom was performed using the SimpleITK library in Python.^24^, ^25^ After segmentation via thresholding, two morphological operations—closing and dilation—were applied in Python using the OpenCV library to improve segmentation and correct errors introduced by the thresholding, especially in the MR images.^26^ To assess the overlap between the segmented volumes, the Dice similarity coefficient (DSC) was calculated following: $DSC=\frac{2\left|X\cap Y\right|}{\left|X\right|+\left|Y\right|}$, where $\left|X\right|$ and $\left|Y\right|$ represent the number of voxels in each set, respectively.

#### 3.5.2 In vivo

The same tools and workflow steps used for phantom analysis were applied for in vivo $\Delta r_{tot}$ displacement quantification. Six landmarks were manually placed around the skull by three observers on three coronal slices and three sagittal on both in vivo CT and MR images (Figure 2c) via 3D‐Slicer. Manual segmentation of three coronal and sagittal slices was executed by three different observers on CT and MR images for DSC calculation. The average of the DSC values obtained by the different observers was performed before and after correction methods.

## 4 RESULTS

### 4.1 Phantom‐based $\Delta B_{0}$ distortion quantification and shimming optimization

**FIGURE 3:**
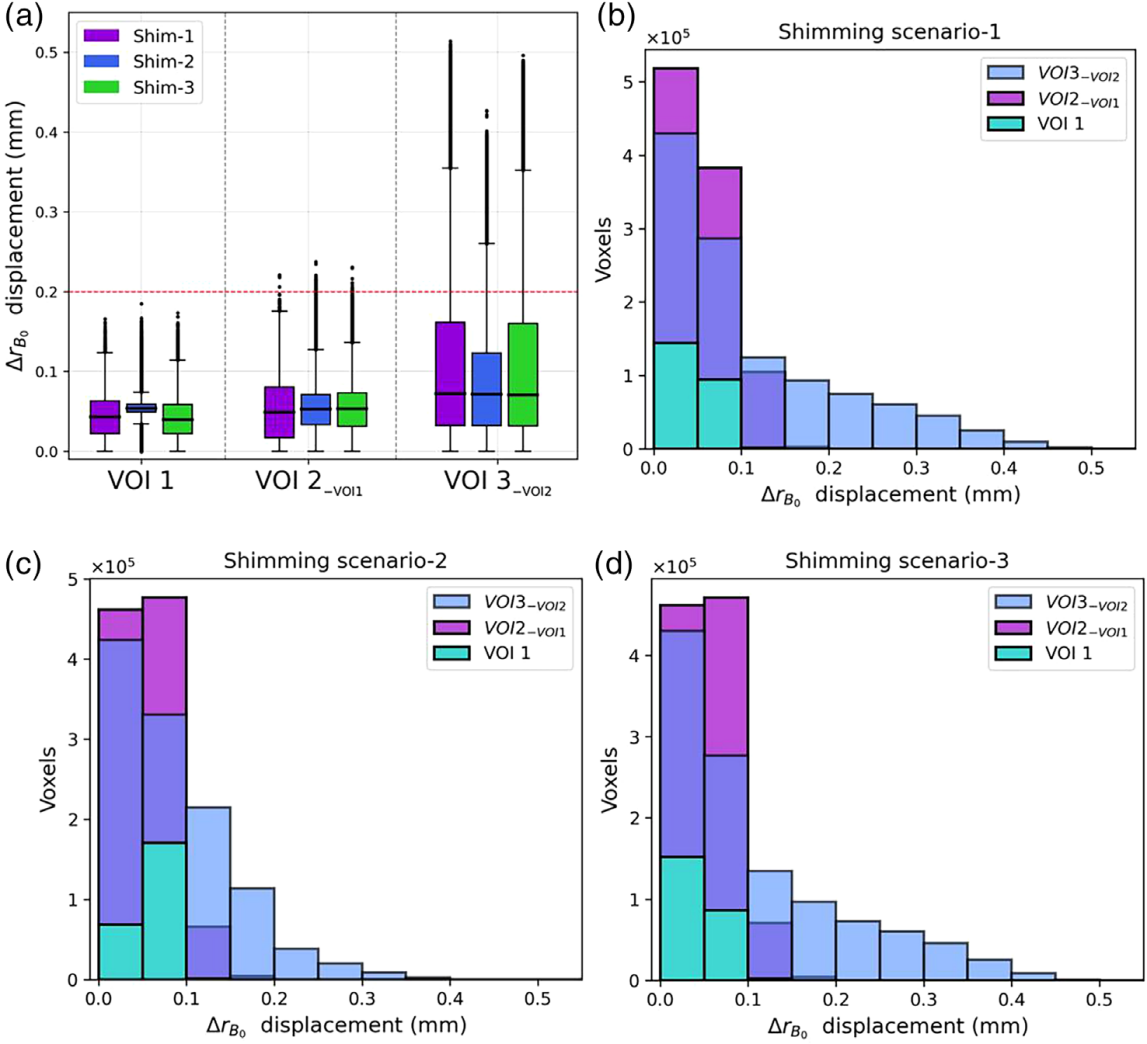
(a) Boxplot showing the distribution of $\Delta r_{B_{0}}$ displacement (mm) in the z‐readout direction for each volume section, left to right from smallest to largest, across all shimming scenarios; in purple Shim‐1, blue Shim‐2, and green Shim‐3. Median values are represented as black bars. The red line indicates the 0.2 mm threshold. In (b) the histogram shows the voxels distributions of each VOI—light green for VOI1, lilac for $VOI2_{-VOI_{1}}$, light blue for $VOI3_{-VOI_{2}}$—based on the $\Delta r_{B_{0}}$ displacement (mm) observed for Shim‐1. Similarly, (c) histogram shows the voxel distribution of $\Delta r_{B_{0}}$ displacement for Shim‐2, and (d) for Shim‐3. VOI, volume of interest.

Shim‐1 and ‐3 required the same acquisition times during the automatic adjustment procedure, whereas Shim‐2 required a pre‐acquired static field map and manual positioning of a shimming volume, resulting in an extra scan time of approximately 16.5 min. A threshold of 0.2 mm displacement was selected as an internal workflow‐specific goal for geometric accuracy. Figure 3a shows the distribution of the $\Delta r_{B_{0}}$ displacement in the z frequency direction across all the voxels of the three static field maps per each VOI and shimming scenario (Section 3.3). In Figure 3b–d, histograms display the distributions of voxels within each VOI according to the displacement $\Delta r_{B_{0}}$ in millimeters encountered under Shim‐1, ‐2, and ‐3, respectively. Most shimming scenarios had maximum $\Delta r_{B_{0}}$ displacements lower than the 0.2 mm threshold within $VOI2_{-VOI_{1}}$ along the z‐axis, excluding the outliers (Figure 3a). At greater distances up to 25 mm ($VOI3_{-VOI_{2}}$), the $\Delta r_{B_{0}}$ displacements increase rapidly to more than 0.3 mm (Figure 3a). However, the majority of voxels were distributed below 0.2 mm displacement (Figure 3b–d). For the largest VOI, both Shim‐1 and ‐3 revealed maximum $\Delta r_{B_{0}}$ displacements (max ± std) of 0.35 ± 0.10 mm and Shim‐2 of 0.26 ± 0.07 mm (Figure 3a), excluding the outliers. Shim‐2 showed the lowest maximum displacement and variation across all VOIs, improving the effect of distortion the most.

### 4.2 Phantom results

**FIGURE 4:**
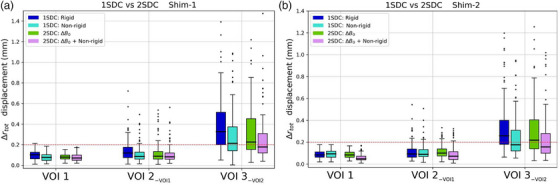
Boxplots illustrating the distribution of $\Delta r_{tot}$ displacement (mm) in phantom images for each volume size after Shim‐1 (a) and Shim‐2 (b). For the 1SDC pipeline, non‐corrected after rigid registration displacements (dark blue) are compared to corrected displacements after non‐rigid registration (light blue). For the 2SDC pipeline, $\Delta B_{0}$‐corrected displacements after rigid registration (green) are compared to $\Delta B_{0}$+non‐rigidly corrected displacement (pink). Median values are represented as black bars. The red line indicates the 0.2 mm threshold. 1SDC, one‐step distortion correction; 2SDC, two‐step distortion correction.

The non‐rigid registration in 1SDC pipeline improved the distortion correction of MR images across all shimming scenarios, particularly in grid regions distant from the VOIs' center, and therefore from the magnet isocenter. In Figure 4, the boxplots of the original and residual $\Delta r_{tot}$ displacements for each VOI size are displayed after rigid registration and correction by comparing Shim‐1 with the best Shim‐2. The displacement increased with VOI size for both shimming scenarios. In this evaluation of the displacements using landmarks, the median values of each dataset are prioritized over maximum values, which are more susceptible to observer‐related uncertainties due to manual errors. After correction, the median $\Delta r_{tot}$ displacement (med ± std) in GRE‐images improved from 0.33 ± 0.27 mm to 0.21 ± 0.24 mm for Shim‐1 and from 0.26 ± 0.25 mm to 0.17 ± 0.19 mm for Shim‐2 in the largest $VOI3_{-VOI2}$ (Figure 4a,b). Within $VOI2_{-VOI1}$, the median displacement was reduced from 0.12 ± 0.09 mm to 0.09 ± 0.08 mm for Shim‐1 and from 0.09 ± 0.08 mm to 0.08±0.07mm for Shim‐2. Considering the best Shim‐2, 100%, 89.7%, and 56.5%—from the smallest to the largest VOI, respectively—of the landmarks showing $\Delta r_{tot}$ displacement post 1SDC correction was within the threshold of 0.2 mm (Figure 4).

**FIGURE 5:**
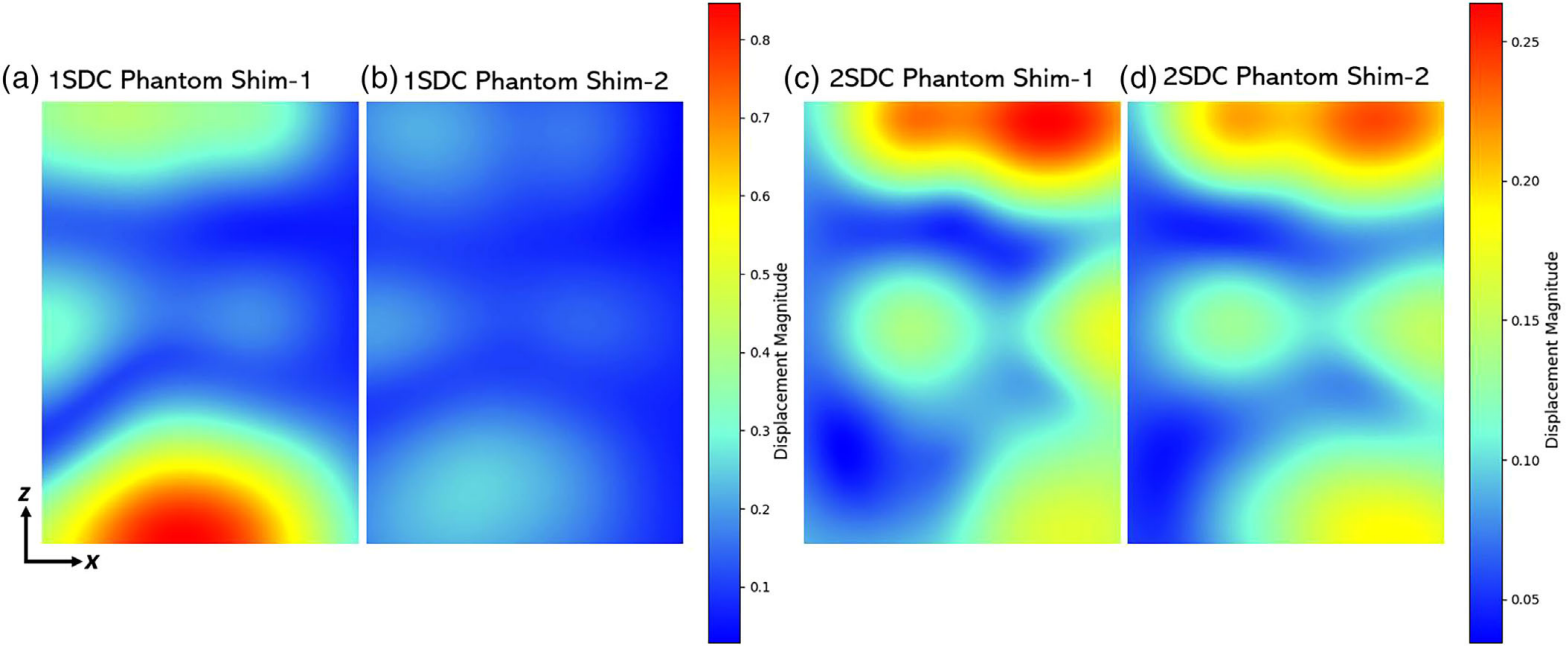
Central coronal slices of the displacement fields generated from non‐rigid correction for Shim‐1 (a) and ‐2 (b) for 1SDC, while (c) and (d) for 2SDC in phantom images. 1SDC, one‐step distortion correction; 2SDC, two‐step distortion correction.

The 2SDC pipeline resulted in a displacement trend equal to that of 1SDC across the different VOIs. In addition, initial $\Delta B_{0}$ correction and following non‐rigid registration for GNL mitigation showed slightly reduced $\Delta r_{tot}$ displacements compared to 1SDC values. For the largest VOI, $\Delta r_{tot}$ decreased from 0.23 ± 0.34 mm to 0.18 ± 0.23 mm for Shim‐1 and from 0.22 ± 0.24 mm to 0.16± 0.21mm for Shim‐2 (Figure 4a,b). Similar to 1SDC results, for smaller volumes within $VOI2_{-VOI1}$, the median displacement reduced from 0.09 ± 0.1 to 0.08 ± 0.07 for Shim‐1 and from 0.1 ± 0.06 mm to 0.07 ± 0.06 mm for Shim‐2. After the full 2SDC correction, 100%, 92.1%, and 59.3% of the landmarks were within the 0.2 mm threshold for Shim‐2 (Figure 4). Overall, Shim‐2 showed lower displacements across all VOIs before and after corrections for both pipelines. Table 1 reports the dice coefficients calculated for the largest VOI obtained before and after the different correction methods for each pipeline and shimming scenario. The results showed a similar tendency of the landmarks displacements. In the 1SDC pipeline, the dice score increased from 0.49 to 0.5 for Shim‐1 and from 0.75 to 0.77 for Shim‐2. For the 2SDC pipeline, the values improved from 0.65 to 0.68 for Shim‐1, and from 0.75 to 0.78 for Shim‐2. Coronal phantom displacement maps, generated post non‐rigid registration, highlight the voxel displacement magnitudes applied to the MR images to unwarp them with the CT images (Figure 5). The displacements strongly increased along the z direction up to 0.85 and 0.25 mm for Shim‐1 and ‐2, respectively, in the 1SDC method. For the 2SDC, Shim‐1 and ‐2 reached maximum displacements of 0.26 and 0.24 mm in the z direction.

**TABLE 1:** Phantom and mouse brain dice scores of CT versus MR segmentation for the different methods and shimming scenarios.

			Shimming scenario	
	Method	Registration	Shim‐1	Shim‐1
**Phantom**	1SDC	Rigid	0.49	0.75
	1SDC	Non‐rigid	0.50	0.77
	2SDC	$\Delta B_{0}$	0.65	0.75
	2SDC	$\Delta B_{0}$+Non‐rigid	0.68	0.78
**Mouse brain**	1SDC	Rigid	0.95	
	1SDC	Non‐rigid	0.84	
	2SDC	$\Delta B_{0}$	0.95	
	2SDC	$\Delta B_{0}$+GNL	0.97	

Abbreviation; 1SDC, one‐step distortion correction; 2SDC, two‐step distortion correction; GNL, gradient non‐linearity.

### 4.3 In vivo results

**FIGURE 6:**
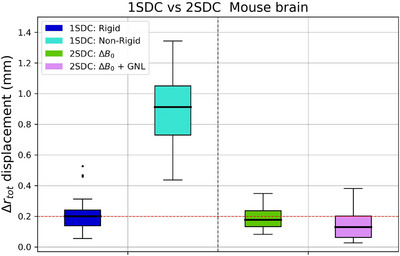
Boxplot showing the distribution of $\Delta r_{tot}$ displacement (mm) in mouse brain image. For the 1SDC pipeline, non‐corrected after rigid registration displacements (dark blue) are compared to corrected ones after non‐rigid registration (light blue). For the 2SDC pipeline, $\Delta B_{0}$‐corrected after rigid registration displacements (green) are compared to $\Delta B_{0}$+GNL‐corrected after the phantom displacement field map application (pink). Median values are represented as black bars. The red line indicates the 0.2 mm threshold. 1SDC, one‐step distortion correction; 2SDC, two‐step distortion correction; GNL, gradient non‐linearity.

Figure 6 illustrates the boxplots of the original and residual $\Delta r_{tot}$ displacements of the mouse brain comparing the distortion correction methods. For the 1SDC, the median $\Delta r_{tot}$ displacement (med ± std) after correction increased from 0.2 ± 0.14 mm to 0.91 ± 0.24 mm. Initially, the highest median displacement was 0.12 mm along the *z*‐axis, with 0.07 mm in *x* and 0.1 mm in *y*. After correction, median displacements significantly increased to 0.24 mm (*x*), 0.81 mm (*y*), and 0.27 mm (*z*). Conversely, in the 2SDC pipeline, the median displacement reduced from 0.18 ± 0.08 mm to 0.13± 0.1 mm post full correction. After $\Delta B_{0}$ correction, the highest median displacement occurred in the *z* direction at 0.09 mm, with *x* and *y* displacements at 0.07 mm each. Following full 2SDC correction, median displacements reduced to 0.06, 0.05, and 0.02 mm along the *x, y*, and *z* axes, respectively.

In Table 1 the in vivo mouse brain dice scores are reported. Results aligned with the trends in landmark displacements: in the 1SDC pipeline, dice scores decreased from 0.95 to 0.84, while in the 2SDC scores improved from 0.95 to 0.97.

## 5 DISCUSSION

To exploit the benefits provided by a UHF‐MR scanner for future preclinical radiation research, system inherent image distortions and relative mitigation strategies needed to be investigated. Novel QA approaches to measuring system‐dependent geometric distortions were mainly tested for clinical MR scanners but require further studies for UHF scanners, predominantly used in preclinical settings. To the authors' knowledge, a 15.2T MR system has to date not been applied to preclinical radiation purposes.

To overcome the small bore diameter of the 15.2T scanner and the RF coil dimension (Section 3.2.1), a customized setup, consisting of a 3D‐printed phantom and holder, was designed to perform the MR measurements while preserving reproducible positioning. For preclinical radiation studies with mice at our facility, the dose grid based on CT images is set to a minimum voxel size of 0.2 mm. For this reason, an isotropic resolution of 0.2 mm was selected for both CT and MR images to ensure comparability and consistency. Phantom and in vivo 3D‐GRE sequences were acquired to verify comparability with the literature data, followed by an assessment of the impact and effectiveness of the applied correction methods.

Pertaining to the first objective, the $\Delta r_{B_{0}}$ increased overall with VOI size, particularly with greater distance along the z readout encoding direction for all shimming scenarios, as reported in clinical QA studies.^7^, ^9^, ^16^ For regions larger than VOI1, achieving complete geometric accuracy within 0.2 mm solely through shimming optimization was unattainable. A significant signal loss artifact, mainly due to $\Delta B_{0}$ (Figure 2c), was evident in MR images ∼15 mm from the VOI center in the lower grid phantom region, restricting FoV acquisition. A similar trend was observed by Baldwin et al. at 3.0T, where the lateral extend of the phantom could not be scanned due to severe inhomogeneities at the bore's edge.^9^ Initial shimming optimization slightly mitigated the artifact, although it could not be fully overcome. Specifically, Shim‐2 improved the homogeneity in the central phantom region but a strong inhomogeneity persisted at the bottom of the image. Shim‐3 was tested to assess if there is a consistent bias due to poor passive shimming that can be ameliorated with higher‐order shims without requiring a $\Delta B_{0}$ map, offering a potentially more time‐efficient protocol than Shim‐2. Nevertheless, Shim‐3 created a homogeneous region around 185 Hz but was not centered in the object and isocenter (Appendix A), which does not allow for a reliable and consistent homogeneity optimization in the target area. This additionally could suggest that the 15.2T Bruker scanner may not be a well‐shimmed system (both passive and active shimming) for radiation therapy purposes. The growing trend of UHF scanners toward higher field strengths—up to 18T—in preclinical research may pose challenges for geometric accuracy in MR images, highlighting the need for robust distortion QA.

Among the scenarios, Shim‐2 resulted in the most effective strategy by exhibiting the lowest maximum $\Delta r_{B_{0}}$ displacement and variability (Figure 3), despite requiring a longer procedure time due to $\Delta B_{0}$ map acquisition (Section 3.3). For the phantom‐based analysis, Shim‐2 involved additional acquisition time to ensure the same resolution and FoV as the GRE images facilitating post‐processing steps. However, for in vivo acquisitions, it is crucial to minimize the scan time. In our in vivo MR protocols, Shim‐2 is widely used, especially in anatomical regions with prominent soft tissue‐air interfaces, where artifacts and $\Delta B_{0}$ distortions can occur (e.g., nasal and ear cavities, lungs, etc.). For the presented mouse brain case, the static field map with 500$\;\mu m^{3}$ resolution was generated with acquisition times <3 min to avoid impacting the total protocol time and the anaesthesia duration. Furthermore, in vivo $\Delta B_{0}$ maps for mouse brains included additional patient‐dependent distortions from susceptibility effects caused by soft tissue‐air boundaries, which were negligible in the phantom analysis due to its homogeneous structure. Therefore, $\Delta B_{0}$ map is needed as a pre‐scan for each patient.

As the second objective, this study demonstrates a substantial minimization in voxel displacement from gradient nonlinearity and static field inhomogeneities through non‐rigid correction (1SDC) on phantom images. Notably, further distortion reduction was achieved by applying the 2SDC pipeline, suggesting that $\Delta B_{0}$ and GNL sequential step correction can be more efficient than solely non‐rigid correction. Specifically, applying only the initial $\Delta B_{0}$ correction alone (2SDC) generally resulted in voxel displacement values that were lower or comparable to those observed after 1SDC correction, especially within $VOI2_{-VOI1}$ region. This was further supported by the displacement field maps (Figure 5), indicating that a more intense correction was performed in Shim‐1 compared to Shim‐2, and dice score coefficients (Table 1). The maximum total distortions in the used dataset generally occurred at the top and bottom corners of the grid phantom (Figures 2, 4, and 5), particularly at the edges of the grid points furthest from the VOIs center and isocenter. This was consistent with the trend observed by O'Callaghan et al., which reported larger displacement values by increasing distance from the magnet isocenter with a 9.4T preclinical system, and other clinical studies.^4^, ^7^, ^8^, ^9^, ^13^, ^15^, ^16^ Distortion correction methods were especially needed in these regions. For small volumes within the size of VOI1, rigid registration that does not involve any additional correction besides shimming optimization was found to be sufficient to obtain voxel‐wise precision within 0.2 mm for Shim‐1 and ‐2 (Figure 4). For larger volumes, distortion correction methods (1SDC or 2SDC) are required. Within $VOI2_{-VOI1}$ regions, it is possible to achieve maximum geometric accuracy of 0.2 mm employing the 2SDC, excluding outliers for selection bias. However, in larger regions further from the isocenter along the z direction, full single voxel accuracy (0.2 mm) is not guaranteed even with the 2SDC correction.

As a final step, the proposed correction methods were tested on in vivo mouse brain MR images at 15.2T representing a more realistic case than the phantom. The typical mouse brain size is about 14 mm (rostral‐caudal)×10 mm (left‐right)×6 mm (dorsal‐ventral), depending on the strain.^27^ This falls between $VOI_{1}$ and $VOI_{2}$, making it a practical example for mitigating distortions in small to medium‐sized regions. Despite the positive results of the 1SDC application on the phantom images and its advantage of correcting different distortions in one step, applying the 1SDC method on in vivo images was less successful. The results showed that non‐rigid correction alone (1SDC) in the brain overcorrected and misaligned the MR image, resulting in larger voxel displacements compared to the solely rigid registration with the CT image (Figure 6).^28^ This discrepancy may stem from the limitations of the non‐rigid registration algorithm (Section 3.4), which proved insufficient to accurately correct MR to CT in vivo images, potentially due to tissue‐air contrast pronounced even more by the higher field of strength. Different non‐rigid registration algorithms could be tested to improve this method and achieve better distortion correction on in vivo images at 15.2T. For this practical case, the 2SDC pipeline showed superior performance, reducing median displacement errors by 85% ($\Delta B_{0}$+GNL correction) than the non‐rigid 1SDC application. Nevertheless, the maximum $\Delta r_{tot}$ displacements in the 2SDC dataset slightly exceeded the 0.2 mm threshold (Figure 6). The Dice scores aligned with the in vivo landmark results (0.97 2SDC vs. 0.84 1SDC) (Table 1), demonstrating consistent results. Therefore, in vivo results demonstrated that even small regions, such as the mouse brain size, imaged at 15.2T require precise correction of geometric distortions for accurate preclinical radiation research. This suggests that a more intense distortion correction might be necessary for larger organs or in regions with strong tissue‐air heterogeneity, such as the abdomen. In addition, this study showed the successful distortion correction of in vivo GNL in brain imaging by applying a displacement map derived from a phantom. This emphasizes that distortions resulting from GNL are sequence‐independent, meaning that any scan acquired on a specific scanner will show consistent GNL‐induced displacements across all spatial directions (*x*,*y*,*z*). For each MR system, independently from the field of strength, it is possible to carry out the proposed phantom QA by obtaining a phantom displacement map, which can be used for GNL correction for different in vivo images effectively.

Although the 2SDC method offers a more targeted and efficient distortion correction, it presents several practical limitations, especially for in vivo applications. First, its implementation requires multiple post‐processing steps, increasing the overall correction time compared to 1SDC (Section 3.4). Second, resolution mismatches between in vivo MR images, $\Delta B_{0}$ maps and phantom displacement maps require precise resampling and cropping to ensure proper alignment with the reference CT. Finally, acquiring a $\Delta B_{0}$ map during the MR protocol is essential for subsequent related distortion correction, which increases scanning time. Especially for radiation research purposes with animal models, it is crucial to account for this extra post‐processing time to prevent delays in image acquisition and treatment plan preparation. Our current in vivo imaging workflow, covering all steps from scanning to organ contouring, takes approximately one day for three subjects, making workflow optimization an important issue. To reduce post‐processing steps in future studies, in vivo anatomical GRE images could be acquired with the same resolution as CT (200$\mu m^{3}$). However, as mentioned in the shimming analysis, in vivo $\Delta B_{0}$ map resolution cannot be adjusted due to the prohibitive scan times of our UHF scanner.

Moreover, the 2SDC approach was specifically designed to correct $\Delta B_{0}$ and GNL distortions in Cartesian sequences, such as GRE and SE, but does not account for distortions in non‐Cartesian acquisitions. In Cartesian sequences, k‐space is sampled by acquiring a single line per excitation along a rectilinear grid with uniform spacing in the frequency and phase encoding directions, leading to linear and predictable voxel shifts. While $\Delta B_{0}$ distortions in Cartesian sequences primarily affect the frequency encoding direction (2), their impact becomes more complex in non‐Cartesian techniques, such as echo planar imaging (EPI), radial, or spiral acquisitions. In EPI, where the entire k‐space is sampled following a single excitation, $\Delta B_{0}$ distortions influence both the frequency and phase encoding directions.^9^, ^19^, ^29^, ^30^, ^31^ These additional phase‐related distortions require dedicated correction strategies, such as the phase difference map technique or the point spread function (PSF) mapping, which need to be integrated into our 2SDC method when extending its application to such sequences.^9^, ^19^, ^29^, ^30^, ^31^

Additionally, non‐Cartesian sequences are also highly susceptible to eddy current effects, which were not explicitly measured in this study so far. Eddy currents, originating from rapid switching of pulsed magnetic‐field gradients, induce secondary time‐varying magnetic‐field gradients and magnetic‐field shifts. These can cause image geometric distortions and ghosting artifacts, including shearing, scaling, and line broadening. As addressed in the literature, eddy current‐induced effects are particularly severe on diffusion‐weighted EPI or short echo‐time localized MR spectroscopy, relying on strong and rapidly switched gradients.^32^, ^33^, ^34^, ^35^ Active shielding and gradient pre‐emphasis strategies are commonly employed in MR systems to compensate for these effects. However, post‐processing methods for specific non‐Cartesian sequences are still being developed to reduce residual eddy current artifacts. As a result, our research has focused on specific correction methods for Cartesian imaging in which eddy currents are considered negligible, rather than on non‐Cartesian.^32^, ^33^, ^34^, ^35^ Cartesian sequences are the current standard for MRI in preclinical radiotherapy, including treatment planning; thus this study prioritized the correction of the most dominant system‐dependent distortions ($\Delta B_{0}$ and GNL) affecting these sequences. Future research will explore the application of our methods to non‐Cartesian sequences and the related impact of eddy currents, further expanding their applicability at UHF.

## 6 CONCLUSIONS

This study established and implemented shimming strategies and QA methods (1SDC vs 2SDC) on a 3D‐printed phantom to mitigate system‐dependent geometric distortions ($\Delta B_{0}$ and GNL) in Cartesian MR images at 15.2T for accurate preclinical radiotherapy. Shimming scenario‐2 is currently being introduced into regular in vivo preclinical protocols in the local laboratory. For preclinical radiation research, the 2SDC method demonstrated superior correction performance, especially for volumes larger than $VOI_{2}$ where single‐voxel accuracy (0.2 mm) proved challenging. The in vivo validation showed that even small FoVs (e.g., mouse brain) need accurate geometric distortion corrections, which was achieved with the 2SDC approach but requires a longer post‐processing time and does not account for distortions on non‐Cartesian sequences (e.g., EPI, radial, spiral). Additionally, in vivo GNL correction was successfully applied using a phantom‐based displacement map for brain imaging by exploiting their sequence‐independent distortion feature. To further assess the effectiveness of these methods, future studies will extend testing to different in vivo organs and FoVs, respectively. Moreover, to improve the performance of the 1SDC method in in vivo applications, new non‐rigid registration algorithms will be explored. Furthermore, optimization of the 2SDC method is envisaged to enhance its time efficiency and suitability for preclinical radiotherapy workflows.

## CONFLICT OF INTEREST STATEMENT

Dietmar Georg serves as a deputy editor for the journal *Medical Physics*.

## Figures and Tables

**FIGURE A1:**
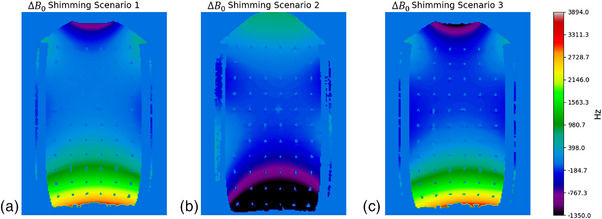
Comparison of shimming scenarios. Central coronal slices of static field maps acquired after Shim‐1 (a), ‐2 (b), and ‐3 (c).

## APPENDIX A

(Figure A1)
